# From metabolic to pathologic complete response: case report of Neoadjuvant Lorlatinib in Stage IIIB ALK-positive NSCLC combined with uniportal VATS

**DOI:** 10.1186/s13019-026-04405-1

**Published:** 2026-06-28

**Authors:** Ruifeng Li, Chudong Wang, Yuxuan Li, Jianyao Zhao, Xinlu Wang, Man Zhang, Shuben Li

**Affiliations:** 1 State Key Laboratory of Respiratory Disease, National Clinical Research Center for Respiratory Disease, National Center for Respiratory Medicine, Department of Thoracic Surgery and Oncology, Guangzhou Institute of Respiratory Health, the First Affiliated Hospital of Guangzhou Medical University, Guangzhou, 510120 PR China; 2 Department of Pathology, the First Affiliated Hospital of Guangzhou Medical University, Guangzhou, China; 3 Department of Nuclear Medicine, The First Affiliated Hospital of Guangzhou Medical University, Guangzhou, Guangdong China

**Keywords:** Non-small cell lung cancer, Neoadjuvant targeted therapy, Pathological complete response, Lorlatinib, Case report

## Abstract

While stage IIIB non-small cell lung cancer (NSCLC) is generally considered unresectable, a subset of patients may benefit from neoadjuvant therapy following comprehensive evaluation. We report a 35-year-old non-smoking female diagnosed with stage IIIB (cT1bN2bM0) adenocarcinoma of the left lower lobe with multiple-station mediastinal lymph node metastasis. Based on multidisciplinary evaluation of multimodality imaging and ALK-positive status, the patient with high tumor burden yet no significant comorbidities received neoadjuvant lorlatinib. The patient achieved a complete metabolic response (CMR) on restaging PET-CT after 10 weeks of lorlatinib, which led to a multidisciplinary decision to undergo uniportal VATS lobectomy. Pathological examination confirmed a pathological complete response (pCR). After an uneventful 5-day hospitalization without complications, the patient remains disease-free at the 12-month follow-up. This is the first report of neoadjuvant lorlatinib followed by uniportal VATS achieving pCR predicted by CMR in stage IIIB ALK-positive NSCLC, given the limitations of short-term follow-up and a single-case design.

## Introduction

Approximately 30% of non-small cell lung cancer (NSCLC) cases are diagnosed at stage III [1], with a subset harboring actionable driver mutations such as ALK fusions. The role of surgery in stage IIIB NSCLC remains controversial, necessitating comprehensive evaluation to identify patients who may benefit from multimodal strategies. In addition, the efficacy of ALK tyrosine kinase inhibitors (ALK-TKIs) in advanced NSCLC and the success of neoadjuvant EGFR inhibition provide a strong rationale for exploring neoadjuvant ALK-TKIs. Despite the extensive use of first- and second-generation ALK-TKIs, their long-term efficacy remains limited by acquired resistance. However, the use of third-generation ALK-TKIs, such as lorlatinib, in the neoadjuvant setting for ALK-positive NSCLC has not been widely reported and may delay the emergence of resistance [2]. Here, we present a rare case of a stage IIIB ALK-positive NSCLC patient who achieved a pathological complete response (pCR) after neoadjuvant lorlatinib, and attempt to utilize complete metabolic response (CMR) as a radiological biomarker to determine treatment duration, aiming to achieve an optimal balance between therapeutic outcomes and cost-effectiveness.

## Case Report

**Fig. 1:**
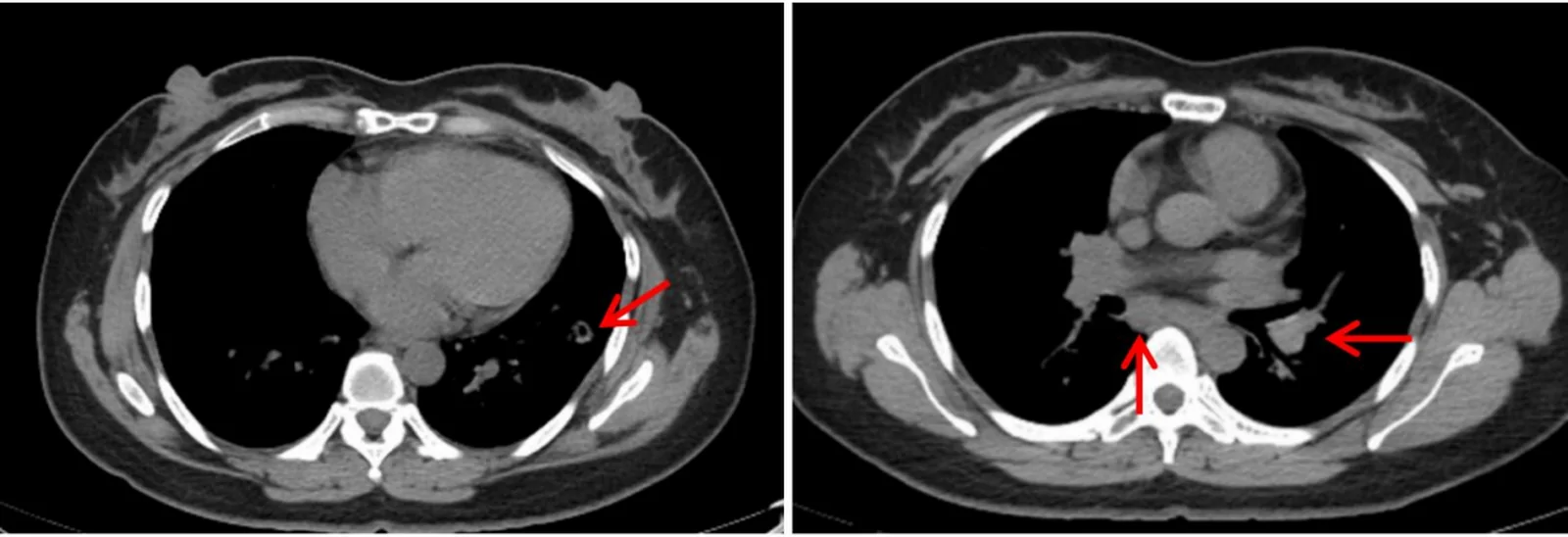
Baseline CT before the inititation of neoadjuvant lorlatinib. Chest CT revealed a nodule in the left lower lobe with hilar and mediastinal lymphadenopathy. CT, computed tomography

A 35-year-old asymptomatic female never-smoker with a documented history of iodinated contrast media allergy, and without significant medical history, presented with an incidentally discovered pulmonary nodule on routine physical examination. Computed tomography (CT) revealed a 1.8 × 1.3 cm solid nodule in the anteromedial basal segment (S7 + 8) of the left lower lobe, accompanied enlarged ipsilateral hilar and mediastinal lymph nodes (Fig. 1).

**Fig. 2:**
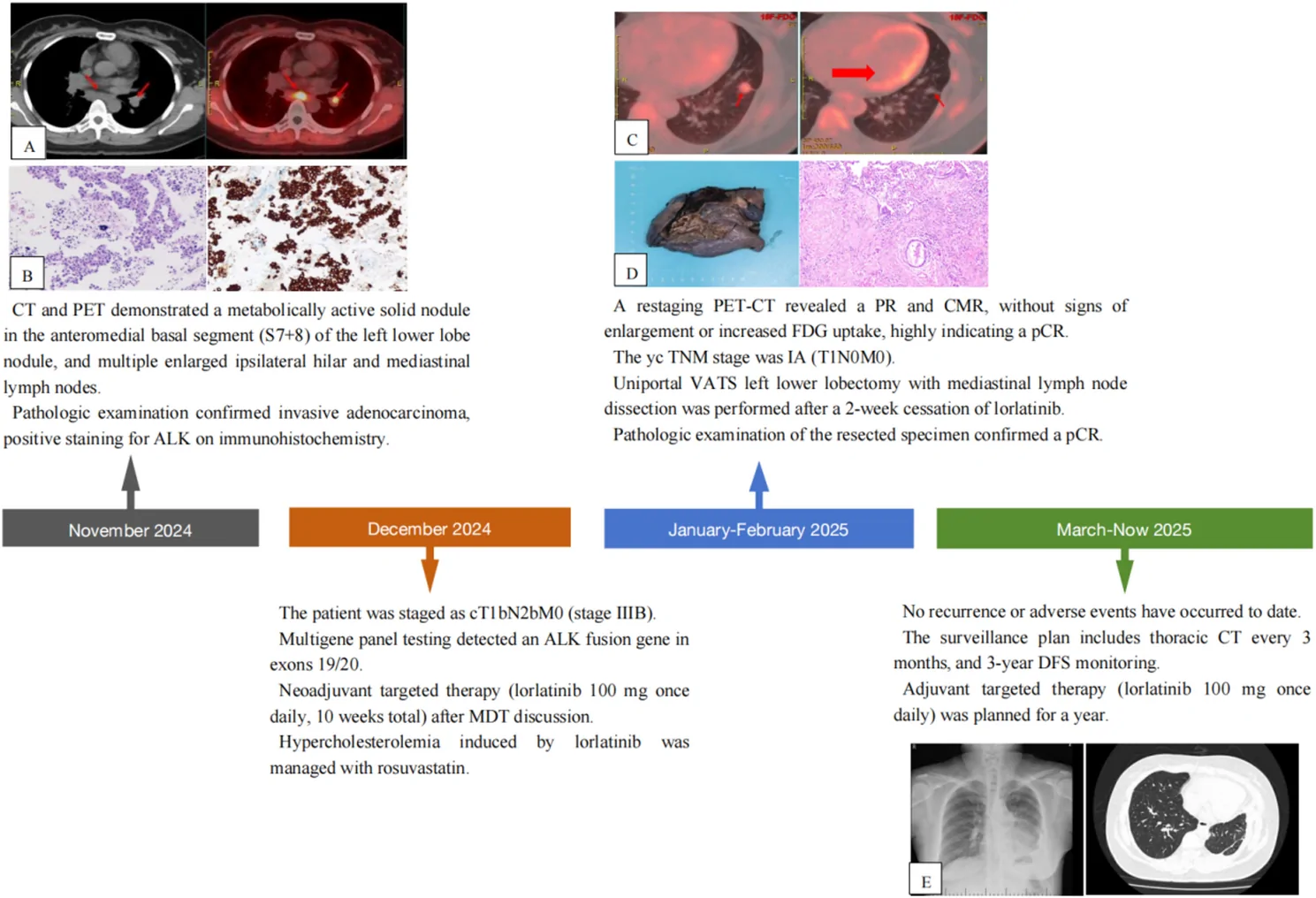
The timeline for the neoadjuvant targeted therapy with radiographic images and pathology results. CT, computed tomography; PET, positron emission tomography; PR, partial response; MDT, multidisciplinary team; CMR, complete metabolic response; VATS, video-assisted thoracoscopic surgery; pCR, pathologic complete response; DFS, Disease-Free Survival

Endobronchial ultrasound-guided transbronchial needle aspiration (EBUS-TBNA) of station 7 lymph nodes was performed. Histopathological examination confirmed invasive adenocarcinoma, showing positive staining for TTF-1, Napsin A, cytokeratin (CK), and ALK protein, with a PD-L1 tumor proportion score of 1%. (Fig. 2B).

Following multidisciplinary team discussion, a comprehensive evaluation was performed. Pulmonary function tests revealed normal lung ventilation. Positron emission tomography-CT (PET-CT) demonstrated increased fluorodeoxyglucose (FDG) uptake within the nodule (SUVmax 3.3) and pronounced fibroblast activation protein inhibitor (FAPI) uptake in the station 7 lymph nodes (SUVmax 11.0), suggesting malignancy with lymph node metastasis in the absence of distant metastasis (Fig. 2A). According to the AJCC/UICC 9th edition staging system, the clinical stage was cT1bN2bM0 (stage IIIB). Multigene testing (quantitative real-time PCR) detected an ALK fusion in exons 19/20, without any co-occurring genetic alterations identified. The specific ALK fusion variant was not further characterized.

After systematic assessment, the patient commenced neoadjuvant therapy with lorlatinib (100 mg once daily). Hypercholesterolemia emerged on day 15 and was managed with rosuvastatin without treatment interruption or dose reduction and no other adverse events were observed. A restaging PET-CT at week 10 demonstrated a partial response (PR): the primary lesion decreased to 1.4 × 0.8 × 0.6 cm without enlargement or increased FDG uptake in the previously involved lymph nodes, consistent with a CMR (Fig. 2C). The post‑treatment clinical stage was ycT1N0M0.

**Fig. 3:**
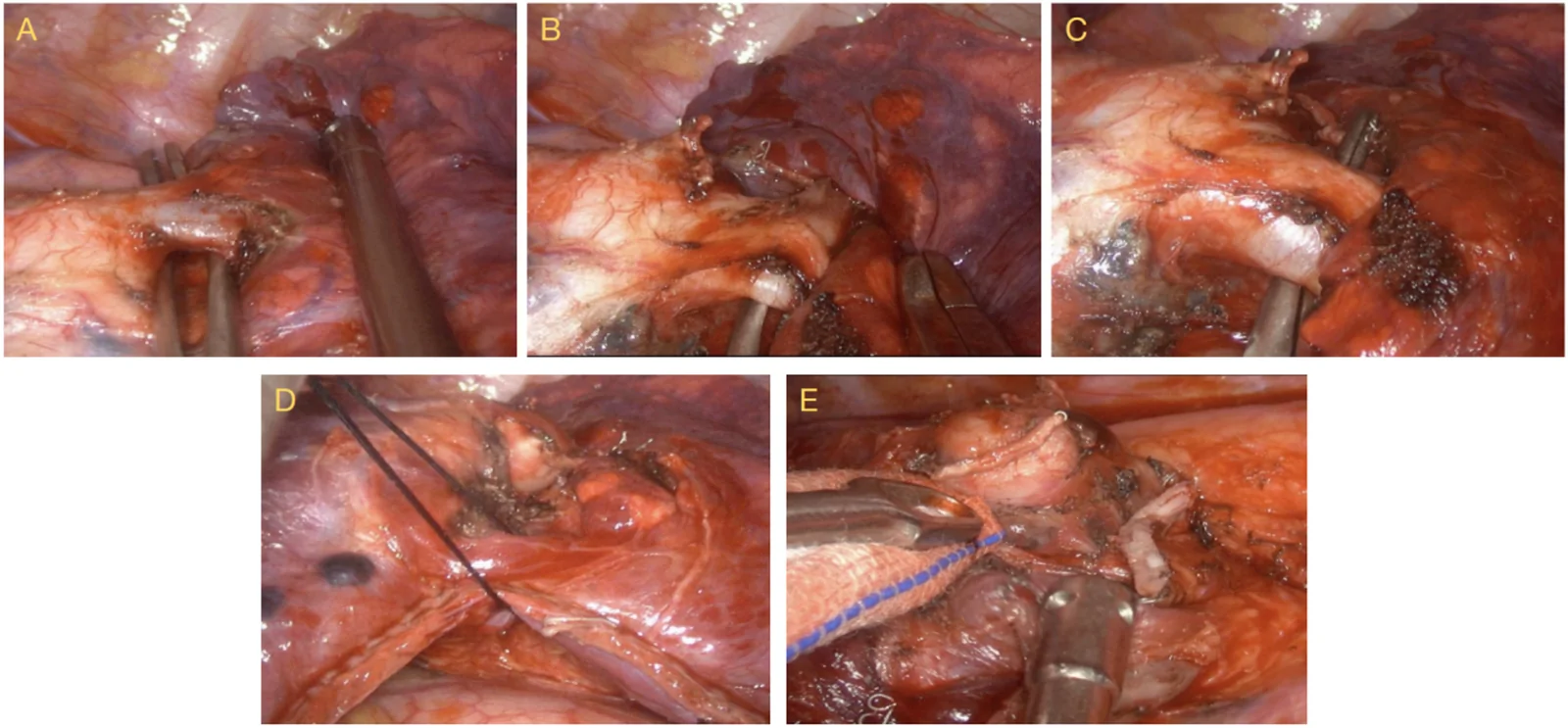
Intraoperative view of uniportal video-assisted thoracoscopic surgery (VATS) left lower lobectomy. (**A-C**) Identification, dissection and transaction of the inferior pulmonary vein (IPV) and lower lobe bronchus (**D**) Ligation of minor vessels and exposure of the bronchial stump. Only mild adhesions and no air leak

After a 10-week course of lorlatinib and 2-week treatment-free interval, uniportal video-assisted thoracoscopic surgery (VATS) left lower lobectomy with mediastinal lymphadenectomy was performed at week 12 (Fig. 3).

Complete surgical resection (R0) was achieved with 20 ml intraoperative bleeding, and only mild adhesions required dissection during the procedure. Pathologic examination confirmed a pathological complete response (pCR), with the tumor bed and lymph nodes showing complete fibrosis and inflammatory replacement (Fig. 2D). The patient was discharged on postoperative day 5 without complications and started on adjuvant lorlatinib (100 mg once daily) with a planned duration of 1 year. The surveillance plan involves thoracic CT every 3 months and 3-year disease-free survival tracking. At the 12-month postoperative follow-up, the patient showed no signs of recurrence or unanticipated events (Fig. 2E). Figure 2 illustrates the timeline of the treatment.

## Discussion

Although surgery is considered the primary treatment modality for NSCLC, curative resection is feasible in only 20–25% of patients at diagnosis [3]. Furthermore, evidence supporting the preoperative use of TKIs, mostly first- and second-generation agents, is limited and postoperative recurrence remains a challenge. Lorlatinib, a third-generation ALK-TKI with broad resistance mutation coverage and proven efficacy in the CROWN trial [4, 5], prompted consideration of its first-line use to suppress acquired resistance and improve survival outcomes. However, in the updated CROWN analysis, grade 3/4 adverse events (AEs) occurred in 76% of patients receiving lorlatinib, most frequently hyperlipidemia [6]. In contrast, no severe AEs were observed in this case during short-term follow-up, and proactive long-term monitoring for hypercholesterolemia was implemented.

In this case, 18 F-FDG PET-CT was used for early response assessment, with complete metabolic response (CMR), defined as 18 F-FDG uptake indistinguishable from surrounding background. Given CMR at 10 weeks, and data from prior trials (at least 9 weeks in NeoADAURA), a 10-week course was adopted. Notably, histopathology remains the gold standard for confirming pCR, while 18 F-FDG PET-CT permits earlier response assessment. The 77.3% SUVmax reduction in primary tumor, which indicated pCR, is consistent with the recent studies [7, 8]. 

As imaging response is not uniformly predictive of pathological response, consolidation surgery is warranted, even when CMR is achieved after 10 weeks of neoadjuvant lorlatinib. It enables resection of occult residual disease, provides pathological confirmation of treatment response, and guides subsequent adjuvant therapy. This rationale is further supported by the NeoADAURA study, which demonstrated a 99% surgery rate and a significant overall survival benefit of surgery after neoadjuvant osimertinib [9], underscoring the role of definitive resection, despite the absence of large phase III trials of neoadjuvant ALK-TKI.

However, Long et al. reported that only 9 of 32 patients with locally advanced NSCLC were eligible for a minimally invasive approach [10], raising concerns that neoadjuvant targeted therapy may complicate subsequent surgery dissection. In contrast, in our case, the uniportal VATS was successfully performed with R0 resection, and only mild adhesions were noted with no air leak, conversion to thoracotomy, and other complications. The drainage tube was removed on postoperative day 3, and the patient was discharged on day 5. These data indicate that neoadjuvant lorlatinib may improve resectability and pCR rate without compromising perioperative safety, and the treatment aligns with Enhanced Recovery After Surgery principles.

Notably, the final analysis of the CROWN trial, with a median follow-up of 64.3 months, demonstrated a 5-year progression-free survival rate of 60% with first-line lorlatinib in advanced ALK-positive NSCLC, with over half of patients maintaining durable disease control beyond 5 years [5]. Similarly, the neoADAURA trial, reported a 12-month event-free survival rate of 95% with neoadjuvant osimertinib monotherapy [9]. These findings confirm that third-generation TKIs can provide sustained efficacy for several years in oncogene-driven NSCLC. Accordingly, the 12-month follow-up period in our case is insufficient to fully evaluate long-term survival, which represents a limitation. In addition, multigene testing (PCR), selected for its cost-effectiveness and expedited results, was limited in the number of genes assessed. Moreover, the inherent limitations of a single-case report preclude generalizability of the findings. In future, we will continue to follow up this patient clinically and radiologically and to validate the sustained benefit of this approach.

## Conclusion

Neoadjuvant lorlatinib may represent a promising and tolerable preoperative strategy for selected patients with stage IIIB ALK‑positive NSCLC, warranting evaluation in randomized controlled trials to define its efficacy, predictive biomarkers of optimal duration, and effects on long‑term survival and surgical outcomes.

## Data Availability

Not applicable.

## Abbreviations

NSCLC: Non small cell lung cancer
ALK: Anaplastic Lymphoma Kinase
EGFR: Epidermal Growth Factor Receptor
CK: Cytokeratin
TKI: Tyrosine kinase inhibitors
VATS: Video-assisted thoracoscopic surgery
PET-CT: Positron emission tomography- Computed tomography
PR: Partial response
pCR: Pathological complete response
CMR: Complete metabolic response
